# Supplementary material for: Comparative multiomics analysis of cell physiological state after culture in a basket bioreactor
Source: Sci Rep. 2022 Nov 23;12:20161. doi: 10.1038/s41598-022-24687-4 (PMC9686226; doi:10.1038/s41598-022-24687-4)
Supplement: Supplementary file 1 — Supplementary Information 1. [file 41598_2022_24687_MOESM1_ESM.zip › raw data/Transcriptomic raw data/6.Enrichment/2.KEGG/TE1vsTC1/ALL/src/csab04630.html]

csab04630


- csab:103237430

- Up regulated genes

103237430(1.35562492669487)

- csab:103224900

- Down regulated genes

103224900(-1.42998371610984)

- csab:103238870

- Up regulated genes

103238870(1.45878079806268)

- csab:103218491

- Down regulated genes

103218491(-2.95858939515048)

- csab:103219268

- Down regulated genes

103219268(-3.50421803072857)
- csab:103223920

- Down regulated genes

103223920(-1.66241974207187)

- csab:103247197

- Up regulated genes

103247197(1.34087353024886)

- csab:103246489

- Up regulated genes

103246489(1.48197213715262)

- csab:103226390

- Up regulated genes

103226390(2.68260263702081)
- csab:103235305

- Up regulated genes

103235305(2.47926657358393)
- csab:103237005

- Down regulated genes

103237005(-1.92426444229972)
- csab:103236305

- Up regulated genes

103236305(1.72662479914397)

Close
